# Supplementary material for: Prediction of transition state structures of gas-phase chemical reactions via machine learning
Source: Nat Commun. 2023 Mar 1;14:1168. doi: 10.1038/s41467-023-36823-3 (PMC9977841; doi:10.1038/s41467-023-36823-3)
Supplement: Supplementary file 1 — Supplementary Information [file 41467_2023_36823_MOESM1_ESM.pdf]

Supplementary Information for

# Prediction of Transition State Structures of General Chemical Reactions via Machine Learning

Sunghwan Choi

Division of National Supercomputing,
Korea Institute of Science and Technology Information,
245 Daehak-ro, Yuseong-gu, Daejeon 34141, Republic of Korea

*E-mail: sunghwanchoi@kisti.re.kr

(a)

| Reaction Index | 700 | 7842 | 9558 |
|---|---|---|---|
| Molecular MAPE(%) | 0.88 | 0. 89 | 0.95 |
| Molecular MAE (pm) | 2.28 | 2.48 | 2.51 |

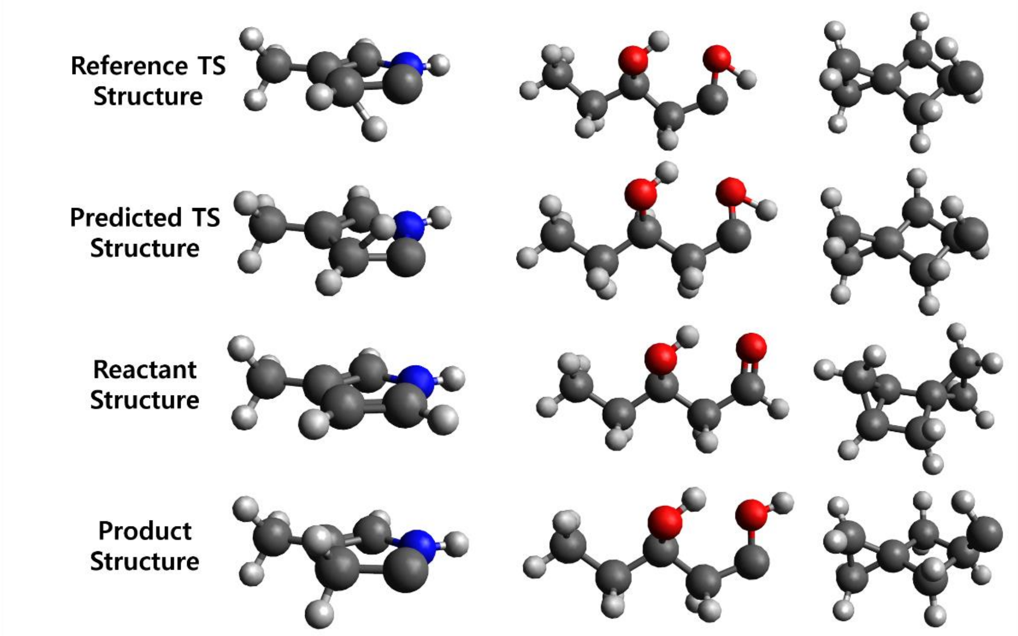


(b)

| Reaction Index | 5028 | 3985 | 2011 |
|---|---|---|---|
| Molecular MAPE(%) | 16.8 | 16.7 | 15.3 |
| Molecular MAE (pm) | 52.3 | 61.2 | 41.9 |

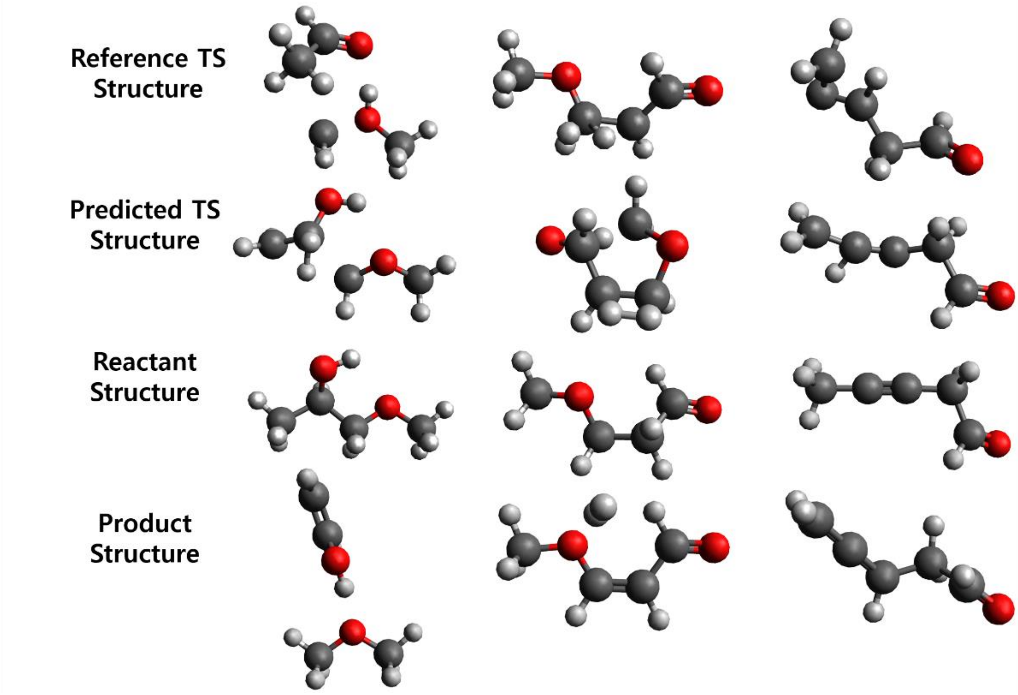


Supplementary Figure 1. Three (a) best and (b) worst inferences for test reactions in terms of molecular mean absolute percentage error (MAPE). The train and test are performed with random spitted subsets of Grambow's reaction database.

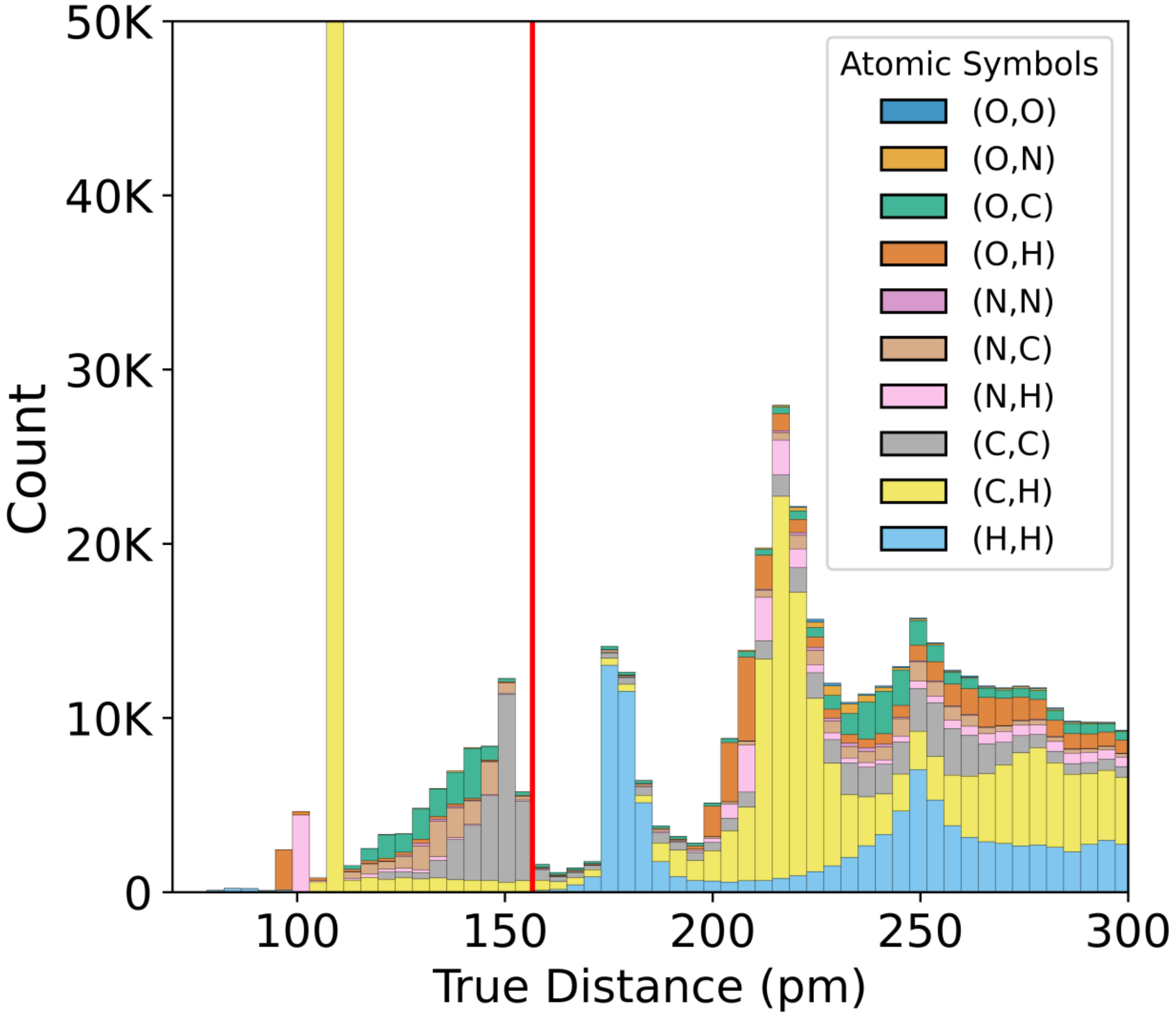


Supplementary Figure 2. Stacked bar chart to represent distribution of all combinations of atomic numbers in Grambow's reaction database. The red vertical line indicate a value of 156.6 pm, which is the criterion for the presence of a chemical bond.

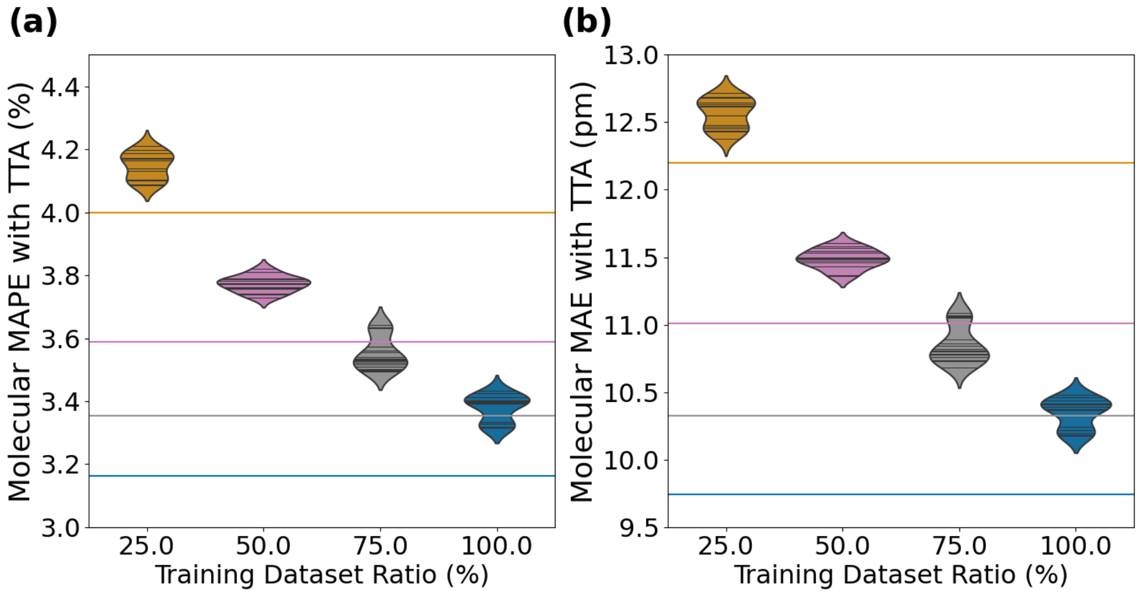


Supplementary Figure 3. Model accuracy in (a) Molecular mean absolute percentage error (MAPE) and (b) Molecular mean absolute error (MAE) according to training dataset size. The solid lines represents the accuracy of ensemble predictions from 15 models (5 epoch ensemble and 3 seed ensemble) The violin plot represents the performance of the single models used in the ensemble. Yellow, pink, grey, blue colors represent the cases trained with 25%, 50%, 75%, and 100% of training dataset, respectively. 500.0, and 0.0 are used for c and c' in Eq. 5 in the main manuscript.

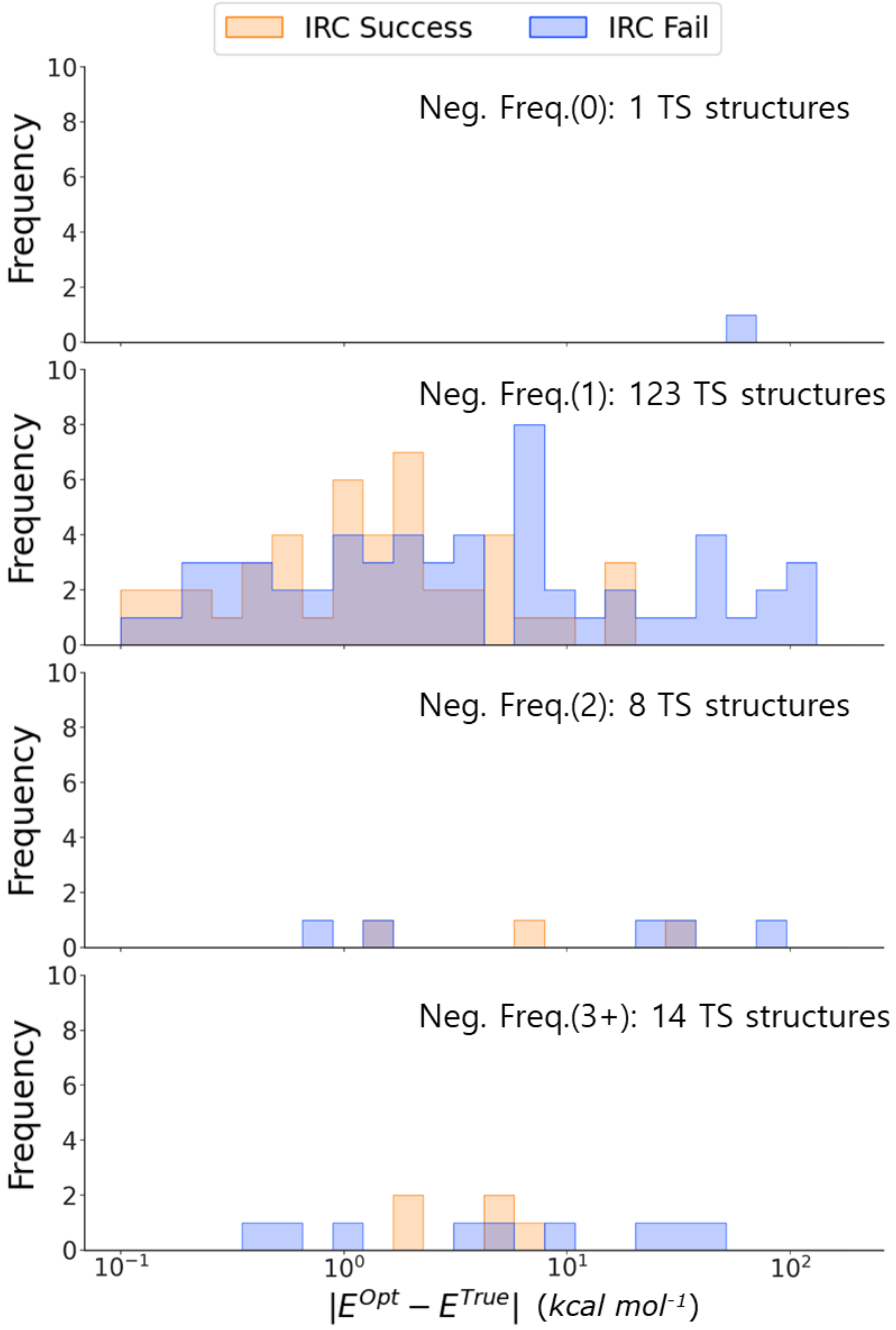


Supplementary Figure 4. Distribution of energy deviations of quantum chemically optimized transition state from machine learning prediction to reference according to number of negative frequencies (Neg. Freq.). Among 126 TS structure that have larger than 0.1kcal/mol energy error, 123 TS structures have one negative frequency which imply the proper saddle point at the potential energy surface. Some cases that have unwanted numbers of negative frequencies are observed. Red and blue bars represents the number of predictions whose geometry yield target reactant and product structures.

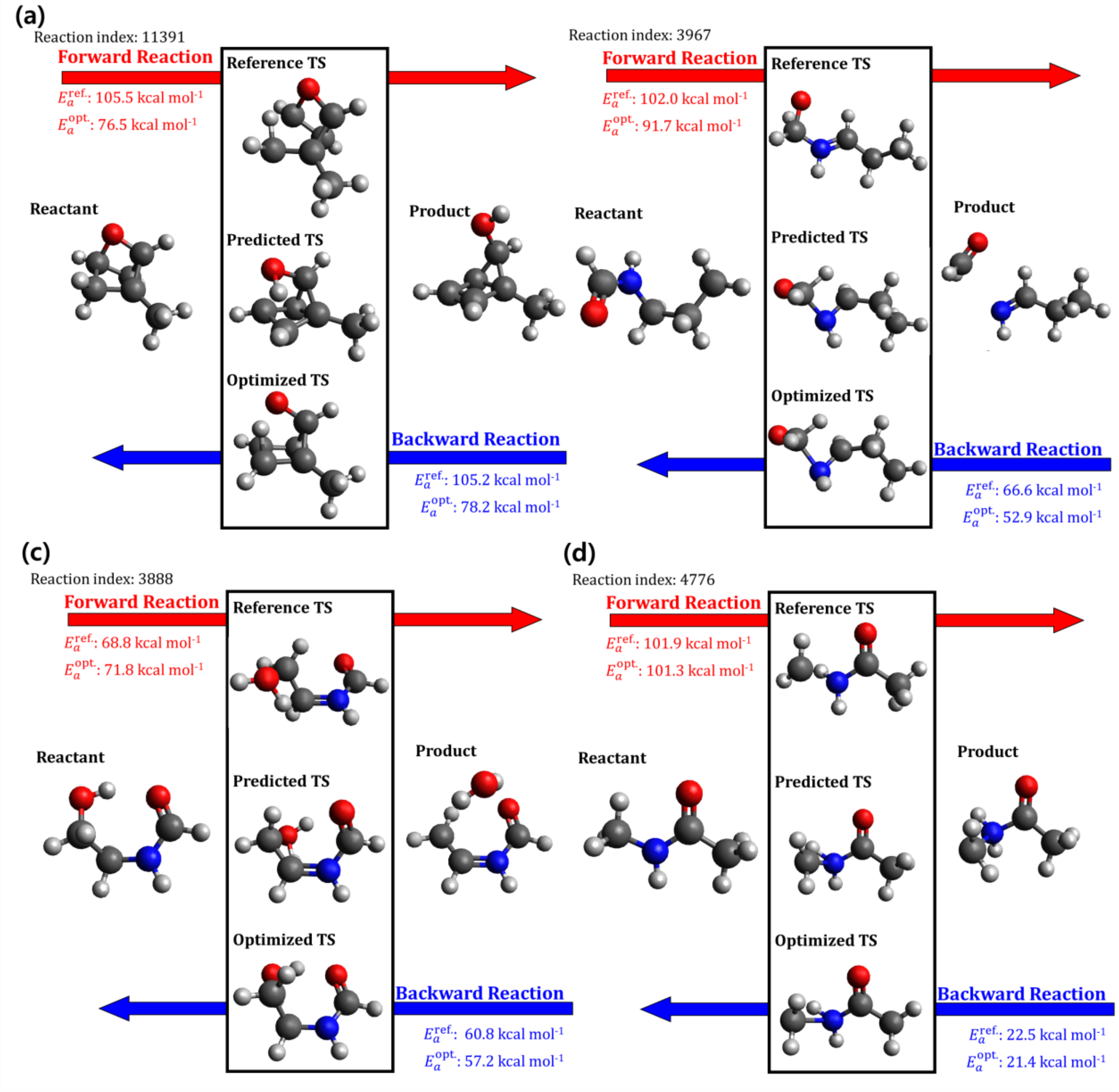


Supplementary Figure 5. Molecular structures and activation barriers of four largest positive error cases among the inferred structures that yielded correct reactant and product structures through intrinsic reaction coordinate calculations. A positive error indicates that the obtained transition state structure is energetically less favorable than the corresponding reference transition state.

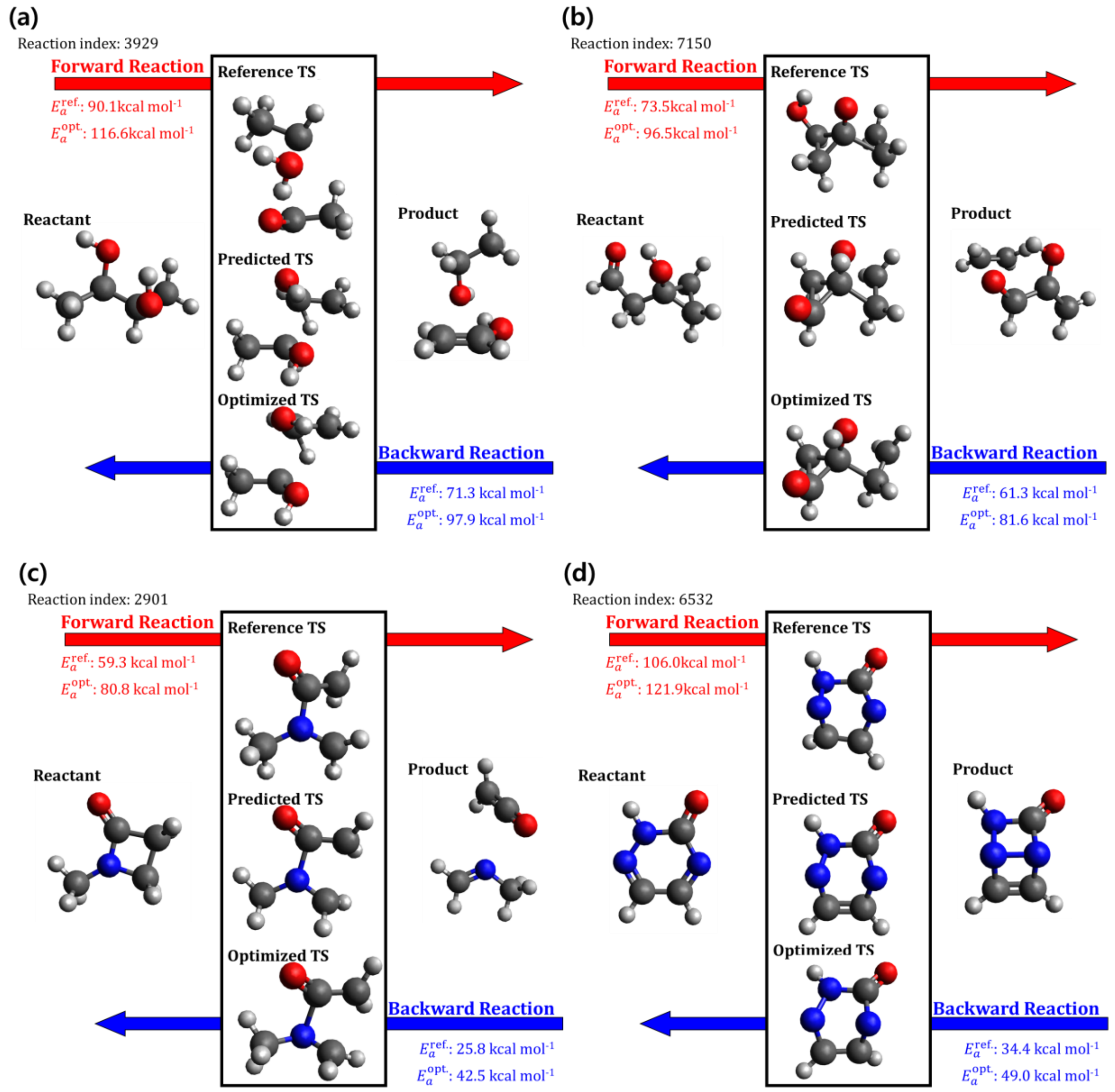


Supplementary Figure 6. Molecular structures and activation barriers of four largest negative error cases among the inferred structures that yielded correct reactant and product structures through intrinsic reaction coordinate calculations. A negative error indicates that the obtained transition state structure is energetically more favorable than the corresponding reference transition state.

Supplementary Table 1. Accuracy of ensemble predictions according to splitting methods.

| TTA | MAPE(%) | | MAE(pm) | |
|---|---|---|---|---|
| | Random splitting | Scaffold splitting | Random splitting | Scaffold splitting |
| Yes | 3.162 | 3.031 | 9.214 | 9.742 |
| No | 3.164 | 3.037 | 9.244 | 9.758 |

Supplementary Table 2. Hyperparameters for model training.

| | |
|---|---|
| Batch size | 80 |
| Learning rate | 0.0002 |
| Gradient clipping value | 1 |
| Precision | 64 |
| Number of GRU Layers | 2 |
| Number of PSI Layers | 2 |
| Dropout for GRU Layer | 0.4 |
| Feature size for atomic numbers | 32 |
| Feature size for interatomic distance | 64 |
| Exponent ($\alpha$) for Gaussian kernel ($Å^{-2}$)† | 10 |
| Grid spacing for centers of Gaussian function (Å) † | 0.1 |

†Embedding of interatomic distances are performed with Gaussian kernel, $f_{ijk} = e^{-\alpha(d_{ij}-g_k)^2}$, where $d_{ij}$, $g_k$, and $\alpha$ are interatomic distance, center of Gaussian, and exponent, respectively. $g_k$ is sampled on equidistant grid on the range of [0, 30.0].

Supplementary Table 3. Molecular mean absolute percentage error (MAPE) and molecular mean absolute error (MAE) of reconstructed TS structures according to ensemble sizes. The lowest error values are bolded. $c$ and $c'$ are weight factors for total loss which are defined in Equation 5 in the main manuscript. When $c'$ is non zero, multi-label learning is performed.

| TTA | hyperparameters | | Molecular MAPE (%) | | | Molecular MAE (pm) | | |
|---|---|---|---|---|---|---|---|---|
| | $c'$ | $c$ | Ensemble Size (5)[1)] | Ensemble Size (15)[2)] | Ensemble Size (90)[3)] | Ensemble Size (5)[1)] | Ensemble Size (15)[2)] | Ensemble Size (90)[3)] |
| Yes | Yes (1.0) | 2000 | 3.263 | 3.168 | 3.162 | 9.999 | 9.749 | 9.742 |
| | | 1000 | 3.266 | 3.169 | | 10.010 | 9.773 | |
| | | 500 | 3.411 | 3.306 | | 10.433 | 10.153 | |
| | No (0.0) | 2000 | 3.233 | **3.112** | | 9.971 | **9.610** | |
| | | 1000 | 3.282 | 3.118 | | 10.064 | 9.632 | |
| | | 500 | 3.348 | 3.162 | | 10.270 | 9.742 | |
| No | Yes (1.0) | 2000 | 3.292 | 3.171 | 3.164 | 10.095 | 9.764 | 9.758 |
| | | 1000 | 3.301 | 3.187 | | 10.120 | 9.839 | |
| | | 500 | 3.466 | 3.316 | | 10.616 | 10.198 | |
| | No (0.0) | 2000 | 3.246 | 3.115 | | 10.018 | 9.627 | |
| | | 1000 | 3.317 | 3.119 | | 10.187 | 9.642 | |
| | | 500 | 3.367 | 3.164 | | 10.333 | 9.758 | |

[1)] Averaging results from 5 best epochs from a single training

[2)] Averaging results from 5 best epochs from 3 independent trainings (3 seeds)

[3)] Averaging results from 5 best epochs from 18 independent trainings (3 seeds and 6 hyperparameters)
