# Supplementary material for: Prediction of transition state structures of gas-phase chemical reactions via machine learning
Source: Nat Commun. 2023 Mar 1;14:1168. doi: 10.1038/s41467-023-36823-3 (PMC9977841; doi:10.1038/s41467-023-36823-3)
Supplement: Supplementary file 3 — Description of Additional Supplementary Files [file 41467_2023_36823_MOESM3_ESM.pdf]

Description of Additional Supplementary Files

Supplementary Data 1: TSNet inference results. The reaction Index refers to the index of Grambow's wb97xd dataset. Absolute error and absolute percentage error is the errors of interatomic distances in pm and percentile, respectively. Atomic numbers represent the atomic numbers of corresponding atomic pairs. BondChange refers to changes in a bond between pairs due to the reaction. The atomic pairs belong to the same reaction are lexicographically ordered according to the order of atomic index of reactant geometry from Grambow's database.

Supplementary Data 2: TSGen inference results. The reaction Index refers to the index of Grambow's wb97xd dataset. Absolute error and absolute percentage error is the errors of interatomic distances in pm and percentile, respectively. Atomic numbers represent the atomic numbers of corresponding atomic pairs. BondChange refers to changes in a bond between pairs due to the reaction. The atomic pairs belong to the same reaction are lexicographically ordered according to the order of atomic index of reactant geometry from Grambow's database.

Supplementary Data 3: Ensemble inference results using the proposed model. The reaction Index refers to the index of Grambow's wb97xd dataset. Absolute error and absolute percentage error is the errors of interatomic distances in pm and percentile, respectively. Atomic numbers represent the atomic numbers of corresponding atomic pairs. BondChange refers to changes in a bond between pairs due to the reaction. The atomic pairs belong to the same reaction are lexicographically ordered according to the order of atomic index of reactant geometry from Grambow's database.

Supplementary Data 4: Molecular mean absolute percentage error (MAPE) and molecular mean absolute error (MAE) of the trained models with different conditions. 90 models with different choices of hyperparameters and random seeds are listed. c and c' are weight factors for total loss which are defined in Equation 5 in the main manuscript. TTA and NLOpt refer test time augmentation and nonlinear optimization, respectively.
